# Role of mucosal-associated invariant T cells in coronavirus disease 2019 vaccine immunogenicity

**DOI:** 10.1016/j.coviro.2024.101412

**Published:** 2024-08

**Authors:** Ali Amini, Paul Klenerman, Nicholas M Provine

**Affiliations:** 1 Translational Gastroenterology Unit, Nuffield Department of Medicine – Experimental Medicine, University of Oxford, UK; 2 Peter Medawar Building for Pathogen Research, Nuffield Department of Medicine, University of Oxford, UK; 3 Pandemic Sciences Institute, Nuffield Department of Medicine, University of Oxford, UK; 4 Centre for Human Genetics, Nuffield Department of Medicine, University of Oxford, UK

## Abstract

Mucosal-associated invariant T (MAIT) cells are an unconventional T cell population that are highly abundant in humans. They possess a semi-invariant T cell receptor (TCR) that recognises microbial metabolites formed during riboflavin biosynthesis, presented on a nonpolymorphic MHC-like molecule MR1. MAIT cells possess an array of effector functions, including type 1, type 17, and tissue repair activity. Deployment of these functions depends on the stimuli they receive through their TCR and/or cytokine receptors. Strong cytokine signalling, such as in response to vaccination, can bypass TCR triggering and provokes a strong proinflammatory response. Although data are still emerging, multiple aspects of MAIT cell biology are associated with modulation of immunity induced by the coronavirus disease 2019 mRNA and adenovirus vector vaccines. In this review, we will address how MAIT cells may play a role in immunogenicity of vaccines against severe acute respiratory syndrome coronavirus 2 (SARS-CoV-2) and how these cells can be harnessed as cellular adjuvants.

**Current Opinion in Virology** 2024, **67**:101412

This review comes from a themed issue on **Mucosal Immunology**

Edited by **Zhou Xing** and **Jean-Michel Sallenave**

For complete overview about the section, refer “Mucosal Immunology (2024)”

https://doi.org/10.1016/j.coviro.2024.101412

1879–6257/© 2024 The Author(s). Published by Elsevier B.V. This is an open access article under the CC BY license (http://creativecommons.org/licenses/by/4.0/).

## Introduction

Mucosal-associated invariant T (MAIT) cells are an important component of the human immune system — although they are often overlooked. In peripheral blood, approximately 3% of T cells are MAIT cells, and they are markedly enriched in the liver, where they represent on average 10% of the T cell pool 1, 2. MAIT cells are readily marked out phenotypically by their possession of a Vɑ7.2 T cell receptor (TCR) chain and expression of high levels of CD161. They can also be defined through their binding to tetramers composed of MR1 bound to 5-OP-RU (5-(2-oxopropylideneamino-6-D-ribitylaminouracil)), their key microbial ligand [3]. Both MR1 and the ligand presentation are conserved in evolution to the extent that MAIT cells can cross-recognise tetramers from the other species 4, 5. This remarkable conservation suggests a critical preserved role in host defence, and there are several examples in mouse models where knockout of MR1, and thus loss of MAIT cells due to a block in thymic selection, leads to susceptibility to specific infections, such as *Legionella* and *Francisella* 6, 7.

As well as this distinctive recognition system through their TCR, MAIT cells possess an unusual ‘innate-like’ capacity to be triggered in a TCR-independent manner. This was first demonstrated in response to toll-like receptor (TLR) triggering [8] and initially found to be a consequence of response to interleukin (IL)-12 and IL-18 in combination [9]. However, subsequent studies have revealed multiple further cytokines can influence this activation, especially type I interferon, tumor necrosis factor (TNF), and IL-15 10, 11, 12. Thus, MAIT cells can ‘sense’ their immediate environment to allow triggering in response to a range of stimuli, including viral infection and sterile inflammation (where cognate antigen is lacking). Furthermore, these cytokine triggers combine with TCR ligation to induce a more profound functional MAIT cell response 13, 14, 15. For example, IL-17 is only produced in substantial amounts following extended combinatorial signalling 16, 17.

As a result of this extreme sensitivity to cytokines and/or TCR ligation, MAIT cells have been found to be activated in a range of infections. This includes a very profound activation in COVID-19, which is linked to both disease activity and outcomes in intensive care settings 18, 19, 20, 21. These data have been reviewed recently elsewhere [22], with MAIT cells activated early after infection with numerous viruses in humans; in particular, MAIT cells are activated early after influenza infection in humans 10, 23 and functionally associated with improved outcomes after influenza infection in mice [24]. There is also emerging data on their role in specific bacterial infections and bacterially derived vaccines, such as Bacillus Calmette-Guerin (BCG) and Tuberculosis (TB), organisms that express the TCR ligand as well as triggering cytokine responses [25]. Although MAIT cell activation has been demonstrated in numerous human infection studies, the overall *in vivo* consequences of this activation remain unknown in many settings.

The impact of COVID-19 and the vaccination programmes against SARS-CoV-2 has been enormous, and a growing literature is accumulating on how MAIT cells may respond to the two major vaccine platforms involved — adenoviral vectored vaccines and mRNA-LNP (lipid nanoparticle) vaccines. Here, we discuss what is known about the role of MAIT cells in response to these vaccines, considering both immunogenicity and reactogenicity, and also what is not known but of significance for future development of these tools.

## Mucosal-associated invariant T cells and the response to adenoviral vectors

**Figure 1:**
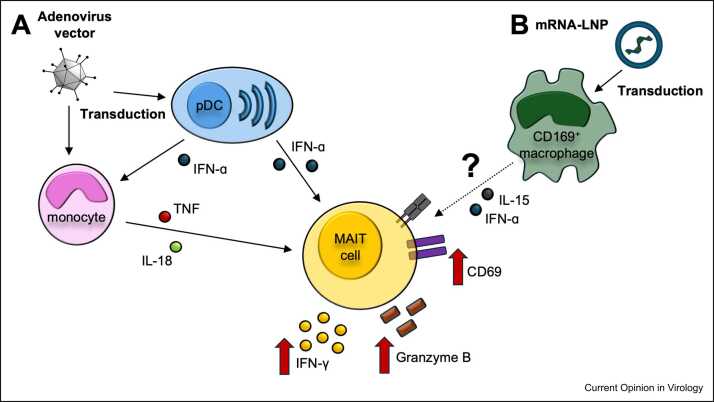
Pathways driving MAIT cell activation by adenovirus vector and mRNA-LNP vaccines. **(a)** Cell populations transduced by adenovirus vectors and the subsequent downstream signalling events that stimulate activation of MAIT cells. Both monocytes and pDCs are transduced by Ad vectors, which stimulate the production of IL-18 and IFN-ɑ by these two cell populations. Cross-talk facilitated by IFN-ɑ also causes TNF release by monocytes. These three cytokines act in concert to activate MAIT cells. **(b)** Cell populations transduced by mRNA-LNP vaccines and subsequent downstream signalling to activate MAIT cells. Many gaps exist in our knowledge. CD169^+^ macrophages are the major transduced population *in vivo*. IL-15 (protein) and type I interferon (transcriptional signature) are both induced by mRNA vaccination. Whether these come from macrophages and are important for MAIT cell stimulation in this context is unknown.

Since MAIT cells have been shown to respond to [10], and protect against [24], virus infection, it has been a question of whether they may similarly positively impact on responses to virally vectored vaccines. Studies in this area have indicated that adenoviruses trigger appropriate cytokines that in combination can strongly activate MAIT cells [26]. The cytokine profile induced is dependent on the adenovirus backbone used in the vaccine. For example, human adenovirus 5 (HuAd5) induces a weak interferon-ɑ and IL-18 response in humans owing to limited transduction of plasmacytoid dendritic cells (pDCs) and minimal activation of the inflammasome in monocytes 26••, 27. In contrast, adenoviruses of other clades, such as ChAdOx1 and Ad26, both of which were used as backbones for SARS-CoV-2 vaccines, strongly trigger these cytokines (Figure 1a). Consistent with this, ChAdOx1 strongly activates MAIT cells *in vitro* in humans, and mice, while HuAd5 has a much more modest effect [26]. The mechanism of MAIT activation by ChAdOx1 is more complex than expected, as exposure of isolated MAIT cells to IFN-ɑ and IL-18 is not sufficient to trigger IFN-γ release, although full triggering is seen if such cytokines are added to mixed PBMCs [26]. The explanation for this is that IFN-ɑ drives TNF production by monocytes, sensing of which is required for full triggering of MAIT cells [26]. Similar requirements are seen for activation of Vδ2^+^ γδT cells [28], a cell type with strong transcriptional and functional parallels to MAITs in humans 29, 30.

**Figure 2:**
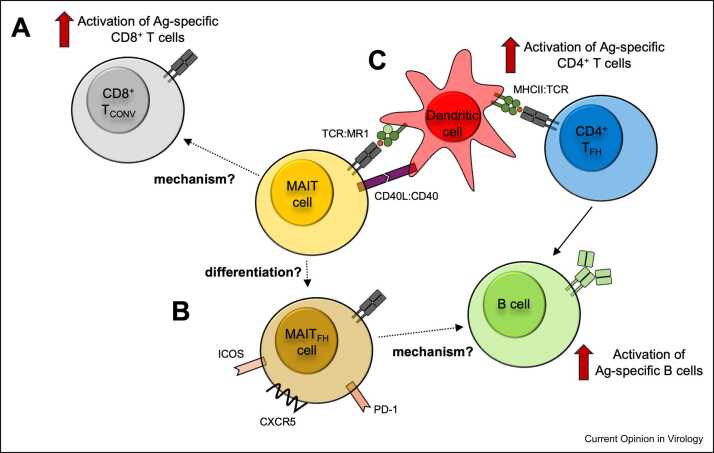
Mechanisms by which MAIT cells can augment adaptive immunity. **(a)** MAIT cells have been shown to enhance conventional CD8^+^ T cell responses induced by adenovirus vector vaccines, but the mechanism is unknown. Furthermore, stimulation of MAIT cells using a TCR ligand also can enhance CD8^+^ T cell function by an unknown mechanism. **(b)** MAIT cells with a phenotype akin to CD4^+^ T_FH_ cells, with high expression of CXCR5, ICOS and PD-1, have been described and associated with help to B cells. PD-1 (programmed cell death protein 1): negative regulator of T cell receptor signalling; ICOS (inducible T cell costimulator): activating receptor; CXCR5 (CXC motif chemokine receptor 5): receptor for the ligand CXCL13, which promotes homing to the B cell follicle. Whether these MAIT_FH_ cells are in a differentiation state or a distinct population of MAIT cells remains unknown. **(c)** Activation of MAIT cells using a TCR ligand can promote DC maturation in a CD40-dependent manner. This in turn can stimulate expansion of CD4^+^ T_FH_ cells, which provide help to B cell responses.

Although these experiments showed a clear mechanism for MAIT activation in response to such vectors, and early MAIT cell activation *in vivo* correlated with subsequent immunogenicity, defining the role of this activation *in vivo* required studies using an MR1 knockout model [26]. Here, CD8^+^ T cell responses to a range of adenovirus vector vaccines (SARS-CoV-2, hepatitis C virus, and the model antigen ovalbumin) showed that an absence of MAIT cells impacted on the induction of conventional, antigen-specific CD8^+^ T cell responses (Figure 2a).

Although these immunogenicity results are consistent in mice and humans, several important questions remain — in particular, how MAIT cells exert their effect is unknown (Figure 2a). In the protection seen against influenza, it was demonstrated that IFN-γ played a significant role [24], but a range of factors, including chemokines, could influence the early events involved in CD8^+^ T cell priming. The impact of MAIT cells on B cell immunogenicity has yet to be explored in a similar way, although their activation has been correlated with antibody titers or memory B cell responses in a nonhuman primate model 31, 32. In other vaccine settings, it has been reported that MAIT cells can adopt a T_FH_-like role 33•, 34, providing a possible mechanistic link (Figure 2b). Finally, the reactogenicity seen with ChAdOx1 and Ad26 vectored vaccines is likely related to the cytokines induced. To what extent MAIT cells may amplify this and contribute to the overall side-effect profile is unknown. Intriguingly, one study examining whole blood from patients vaccinated with Ad26 found a transient signature for MAIT cell activation after prime, similar to MAIT cell activation observed after ChAdOx1 [26], that was reduced in association with antivector neutralising antibodies at boost [35]. In patients with vaccine-induced thrombosis with thrombocytopenia syndrome, transcriptional signatures for activation of coagulation, platelets, and MAIT cells were present 2 weeks after vaccination, which may implicate excessive MAIT cell responses in toxicity [35]. This hypothesis requires formal experimental investigation.

## Mucosal-associated invariant T cells and response to mRNA vaccines

In contrast with Ad vectors, the innate response induced by mRNA vaccines is much more limited — this is part of their design based on replacement of uridine with pseudo-uridine and removal of double-stranded RNA intermediates to minimise innate triggering 36, 37. Thus, one would predict that the activation of MAIT cells during priming would be similarly limited. Although published data have not directly addressed this in the same way as described above for Ad vectors, there are several studies which indicate that this is the case *in vivo* — but the situation is markedly different after a second ‘boosting’ vaccination.

The first study examining MAIT cells explicitly in the context of mRNA vaccination found that baseline characteristics predict BNT162b2-induced adaptive immunity [38]. Prevaccine MAIT cell frequency as a fraction of peripheral blood mononuclear cells (PBMCs) positively correlated with BNT162b2-induced activation-induced marker positive (AIM^+^) CD4^+^ T cell and antispike antibody responses, whereas MAIT cell activation assessed by CD69 expression negatively correlated with these measures of adaptive immunity [38]. Another study found that baseline counts of granzyme B-expressing (GzmB^+^) MAIT cells — normally low in the absence of activation — were inversely associated with spike-specific antibody responses 4 weeks after boost (as measured by pseudoneutralisation titres) [39]. Mechanistically, tonic MAIT cell activation prevaccination might result in desensitisation to further activation, with differences in the abundance and sensitivity potentially impacting on their role as a cellular adjuvant. Alternatively, these associations may simply reflect the sensitivity of MAIT cell activation as a biomarker of deleterious comorbidity-associated inflammation; for example, increased MAIT cell activation is observed in obese patients 40, 41, who also mount substantially lower antibody responses to SARS-CoV-2 vaccines [42].

The main evidence for MAIT cell activation by mRNA vaccines comes from transcriptional studies of human blood. Although nonspecific, a whole blood signature analogous to MAIT cell activation after ChAdOx1 immunisation has also been observed after boost with BNT162b2 and mRNA-1273 in particular [35]. Another study examining peripheral blood MAIT cells by single-cell RNA sequencing (scRNA-seq) before and 3 days after BNT162b2 vaccination found signatures of proliferation and TNF expression, which correlated with spike-specific antibody responses [43]. The same group found enhanced activation, proliferation, and effector potential in analogous cytokine-responsive Vδ2 + γδT cells after BNT162b2 vaccination, with increased transcriptional signatures of effector responses (*IFNG*, *TNF*, and *XCL2*), and memory-associated genes (*IL7R*, *TCF7*, *GATA3*, *EOMES, CXCR3*) after booster vaccination [44].

In terms of how MAIT cells could be activated (Figure 1b), CD169^+^ macrophages are the main population transduced by mRNA vaccines from animal studies [45]. Cytokines, which can activate MAIT cells, such as IL-15, are secreted within 24 hours of mRNA vaccination [46]. Surprisingly, transcriptional evidence for type I IFN production is also present after BNT162b2, albeit only femtomolar concentrations have been detected in plasma, so it is unclear to what extent this contributes [47]. Unusually, two studies have also found MAIT cells amongst spike-peptide responsive T cells in PBMCs of mRNA-vaccinated individuals 48, 49. Although potentially an *in vitro* artefact of the AIM assay, one might envisage similar MAIT cell activation to translated spike protein *in vivo* after mRNA vaccination.

The potential consequences of such activation are unclear. Takano et al. identified a population of CD56^+^ ‘NKT-like’ cells that were associated with both reactogenicity and neutralising antibody titres after a second dose of BNT162b2 [50]. Expression of CD69 amongst these ‘NKT-like’ cells after BNT162b2 boost was found to correlate with the severity of systemic symptoms and plasma IFN-γ. Furthermore, the degree of ‘NKT-like’ lymphopenia after mRNA booster vaccination correlated with spike-specific neutralising antibody titres after 4 weeks. Given a large fraction of MAIT cells express CD56, they likely comprise a proportion of the CD56^+^ T cells described in this study — however, MAIT cells were not specifically identified. The association of GzmB^+^ MAIT cells 1 day after boost with spike-responsive CD8^+^ T cells may further implicate MAIT cells functionally in adaptive immune responses to RNA vaccines [39].

Overall, these correlative data in human cohorts suggest MAIT cells have a role in mRNA vaccine-induced immunity. However, several critical unknowns regarding MAIT cells and mRNA vaccines are as follows: (1) Given the engineering of mRNA vaccines to avoid triggering innate cytokine pathways, how are MAIT cells activated? (2) Why is this activation more pronounced after a second vaccine dose? and (3) What role do MAIT cells directly have on the immunogenicity of mRNA vaccines? Given that MAIT cells are sensitive to cytokines, such as TNF, which are also produced by adaptive immune cells, one could hypothesise that effector memory T cells at boost could directly regulate MAIT cells. Further studies will need to explore the relevance of adaptive immunity to MAIT cell activation and downstream effector functions.

## Mucosal-associated invariant T cells and other vaccine approaches for SARS-CoV-2

Although inactivated vaccines were not as widely used globally as mRNA or adenovirus-based vaccines, they still had an important role in the response to the pandemic, and similarly, the role of MAIT cells has been addressed. Using a scRNA-seq approach, three donors were longitudinally characterised after an initial 4-week boosting interval followed by a longer 32-week interval until the third dose [51]. Interestingly, expansion and differentiation of MAIT cells were seen over time, indicating some antiviral sensing was driving their activation *in vivo* [51]. The consequences of this for immunogenicity are not known.

The approaches above harness the adjuvant potential of MAIT cells as bystanders activated by vaccine-induced innate responses. However, given our knowledge of their biology, it is possible to develop vaccines that trigger MAIT cells in a more targeted way. This has been attempted by Rashu et al., who combined 5-OP-RU administration with either a conventional influenza vaccine or a SARS-CoV-2 vaccine based on recombinant vesicular stomatisis virus (VSV) [52]. Irrespective of route (intraperitoneal, intranasal, and intramuscular), the exposure to 5-OP-RU together with a viral vaccine led to massive expansion of MAIT cells *in vivo*, as has been seen previously with 5-OP-RU and TLR ligands 6, 14, pointing to the potency of combined TCR and cytokine signalling. This may have been important in providing additional protection against a subsequent viral challenge. However, importantly, boosting MAIT cells (in a B6-CAST mouse model, which has already enhanced MAIT cell frequencies at baseline compared with a conventional C57BL/6 mouse [53]) was also accompanied by a strong impact on antiviral CD8^+^ T cells, which could provide a long-lived and specific protection against a severe challenge (Figure 2a). These data suggest that providing a MAIT TCR ligand substantially changes the immunological profile of a viral-based vaccine, resulting in multiple potentially protective effects. For example, MAIT TCR ligation induces unique tissue repair and type 17 immune responses, beneficial in mucosal immune responses, and combination with antiviral cytokines would be expected to increase the magnitude and breadth of tissue MAIT cell effector functions 13, 14, 15, 16.

An alternative approach is to combine a MAIT cell ligand with a protein vaccine, as tested by Pankhurst et al. [54]. Here, a combination of 5-A-RU and methylglyoxal was used (to provide precursors for the unstable 5-OP-RU ligand) as an adjuvant for SARS-CoV-2 spike and influenza targets. Intranasal administration of these adjuvants led to higher levels of protective IgG and IgA antibodies against these antigens including improved protection against challenge. The importance of mucosal administration is unclear, as similar approaches using intraperitoneal immunisation were protective without increasing peripheral antigen-specific IgG [52]. Interestingly, MAIT cell activation was associated with expansion of vaccine-specific CD4^+^ T_FH_ cells, and this was in turn dependent on CD40L-mediated activation of conventional dendritic cells in the MAIT-vaccinated mice (Figure 2c). Thus, MAIT cells here are providing an adjuvant effect for B cells but indirectly through the expansion of CD4^+^ T cells (a feature not observed in the adenovirus system). As MAIT cells also directly promote B cell differentiation and mucosal IgA production after *Vibrio cholera* infection [33], potentially through a T_FH_-like population enriched in mucosal lymphoid organs, future studies need to explore these cells as important targets for protective mucosal vaccines more broadly.

Although MAIT cell activation in combination with mRNA has yet to be tested, a hint of its potential comes from a study of invariant NKT (iNKT) activation in an mRNA model [55]. Here, malaria protection through liver T_RM_ (resident memory T) cells can be induced by irradiated sporozoites but not by conventional mRNA vaccination alone. However, addition of an iNKT ligand into the vaccine protocol led to induction of a protective T_RM_ response in mice. The prototypic iNKT ligand ɑ-galactosylceramide was not suitable for this, and a modified ligand was required. As above, a role for CD40L interactions with DCs was required for the induction of the protective T cell phenotype. Potentially, in humans, recruitment of MAIT cells via combination with a ligand could have a similar impact on T cell immunogenicity and protection, given the substantial overlap in biology between iNKT and MAIT cell populations (and much higher frequencies in humans) [56].

## Conclusions

Several studies in humans and mice indicate the power of MAIT cells in effectively adjuvanting a range of different vaccine types. Natural activation of MAIT cells by virally vectored vaccines such as adenoviruses can provide important early amplification signals that contribute to T cell and potentially also B cell immunogenicity. It is possible this occurs through the activation of antigen presenting cells (APCs), such as DCs, as has been shown in TCR-triggered models. TCR triggering of MAIT cells by provision of ligand in combination with viral or protein vaccines can substantially amplify these effects. Hints suggest similar processes occur following mRNA vaccination, but this needs further clarification. Conversely, the (inflammatory) effector functions of MAIT cells may also contribute to excessive inflammation after mRNA and Ad vector vaccination, potentially relevant to the tolerability and safety of schedules. Defining further the mechanisms underpinning both activation and downstream functional impacts will improve future approaches in this area.

## Funding

PK is supported by a Wellcome (United Kingdom) Senior Fellowship [222426/Z/21/Z], the NIH (United States of America) (U19 I082360), the 10.13039/501100013373NIHR Oxford Biomedical Research Centre (United Kingdom), an NIHR (United Kingdom) Senior Fellowship, and the University of Oxford (United Kingdom) NDM COVID-19 emergency relief fund. NMP is supported by a Wellcome (United Kingdom) Career Development Award [227217/Z/23/Z]. AA is supported by an NIHR (United Kingdom) BRC post-doctoral award.

## Declaration of Competing Interest

Nicholas M Provine reports a relationship with Infinitopes that includes consulting or advisory. Paul Klenerman reports a relationship with UCB that includes consulting or advisory. Paul Klenerman reports a relationship with Biomunex that includes consulting or advisory. Paul Klenerman reports a relationship with AstraZeneca PLC that includes consulting or advisory. Paul Klenerman reports a relationship with Infinitopes that includes consulting or advisory. If there are other authors, they declare that they have no known competing financial interests or personal relationships that could have appeared to influence the work reported in this paper.

## Data Availability

No data were used for the research described in the article.
